# A comprehensive annotation dataset of intact LTR retrotransposons of 300 plant genomes

**DOI:** 10.1038/s41597-021-00968-x

**Published:** 2021-07-15

**Authors:** Shan-Shan Zhou, Xue-Mei Yan, Kai-Fu Zhang, Hui Liu, Jie Xu, Shuai Nie, Kai-Hua Jia, Si-Qian Jiao, Wei Zhao, You-Jie Zhao, Ilga Porth, Yousry A. El Kassaby, Tongli Wang, Jian-Feng Mao

**Affiliations:** 1 Beijing Advanced Innovation Center for Tree Breeding by Molecular Design, National Engineering Laboratory for Tree Breeding, Key Laboratory of Genetics and Breeding in Forest Trees and Ornamental Plants, Ministry of Education, College of Biological Sciences and Technology, Beijing Forestry University, Beijing, 100083 China; 2 College of Big data and Intelligent Engineering, Southwest Forestry University, Yunnan, 650224 China; 3 Départment des Sciences du Bois et de la Forêt, Faculté de Foresterie, de Géographie et Géomatique, Université Laval Québec, Québec, QC G1V 0A6 Canada; 4 Department of Forest and Conservation Sciences, Faculty of Forestry, The University of British Columbia, 2424 Main Mall, Vancouver, BC V6T 1Z4 Canada

**Keywords:** Mobile elements, Data mining

## Abstract

LTR retrotransposons (LTR-RTs) are ubiquitous and represent the dominant repeat element in plant genomes, playing important roles in functional variation, genome plasticity and evolution. With the advent of new sequencing technologies, a growing number of whole-genome sequences have been made publicly available, making it possible to carry out systematic analyses of LTR-RTs. However, a comprehensive and unified annotation of LTR-RTs in plant groups is still lacking. Here, we constructed a plant intact LTR-RTs dataset, which is designed to classify and annotate intact LTR-RTs with a standardized procedure. The dataset currently comprises a total of 2,593,685 intact LTR-RTs from genomes of 300 plant species representing 93 families of 46 orders. The dataset is accompanied by sequence, diverse structural and functional annotation, age determination and classification information associated with the LTR-RTs. This dataset will contribute valuable resources for investigating the evolutionary dynamics and functional implications of LTR-RTs in plant genomes.

## Abstract (summary)

**Table:** 

Measurement(s)	LTR_retrotransposon • genome
Technology Type(s)	bioinformatics method • digital curation
Factor Type(s)	Species
Sample Characteristic - Organism	Rhodophyta • Chlorophyta • Bryophyta • Tracheophyta

Machine-accessible metadata file describing the reported data: 10.6084/m9.figshare.14684877

## Background & Summary

Transposable elements (TEs) are mobile DNA sequences that can move, propagate, and integrate into new positions in the host genomes, and which are ubiquitous in nearly all living organisms^1,2^. All TEs manage to increase their copy number via transposition processes. Depending on the mechanism used for transposition, TEs can be divided into two classes: Class I retrotransposons, which commonly transpose through ‘copy-and-paste’ mechanism of a transcribed RNA intermediate and Class II DNA transposons that move via a ‘cut-and-paste’ mechanism that mobilizes the DNA directly^3^. TEs are often considered as “junk DNA” because of their continuous amplification and potential impairment on the host gene function^4^. However, recently, numerous studies clearly indicated that TEs play a major role in reshaping genome structure through chromosomal rearrangements, gene capture, movement, and exon shuffling^2,5–7^, in creating mutagenic and regulatory variation through their insertion within or near genes^8,9^, and in creating additional genetic diversity underlying species adaptation and evolution^10,11^. Hence, knowledge of their impact on the structure, function and evolution of plant genomes is a priority in the field of genomics.

Among class I elements, the long terminal repeats retrotransposons (LTR-RTs), have been observed to be the most abundant TE component of plant genomes^12,13^, contributing up to 70% of the plant genome size, as reported in maize^14^, wheat^15^, or sugar pine^16^. Moreover, these elements have been considered to be the major source for the observed extensive genome variation of flowering plants, along with polyploidization^17^. In addition, epigenetic silencing of LTR-RTs can affect their impact on major fitness-related traits, including flowering time, a key process of plant life cycle^11,13^. TE-genome wide association studies (TE-GWAS) uncovered that the insertion of LTR-RTs is associated with grain width in rice and fruit weight in tomato^18,19^. LTR-RTs also show unique patterns of development or environment regulation. For instance, maize transcripts *Opie-1* element^20^, barley *BARE-1*^21^, and soybean *SIRE-1*^22^ have been detected primarily in roots, leaves, and seedlings, respectively. Therefore, understanding the molecular causes of genome evolution is of utmost importance, so that the mechanisms regulating LTR-RTs are established, as well as the importance of their transcription to host biology is also better known.

An autonomous LTR-RT that bears all features essential for retrotransposition is composed of two nearly identical LTR sequences which are flanked by target site duplications (TSDs) of usually 4–6 bp^13,23^. In some species, small palindromic motifs at the 5′ and 3′ end of the LTRs are observed^24^. The internal region contains open reading frame, *Gag-Pol*^2,25^. *Gag*, a gene that encodes a polyprotein comprising subcomponents of the virus-like particle (VLP) is involved in the maturation and packaging of retrotransposon RNA, *Pol* products that encode protease (PR), reverse transcriptase (RT), RNase H (RH), and integrase (INT) that are involved in the synthesis of retrotransposon DNA and integration into the host genome^13^. Based on the order of RT and INT in *POL*, LTR-RTs are classified into Gypsy and Copia superfamilies^26^, which are further divided into an enormous number of lineages according to phylogenetic analysis of the polyprotein domains. Usually, plants’ Copia retrotransposons are sub-classified into Ivana (Sirevirus/Oryco), Osser (hemivirus), Bianca and SIRE^27–30^, while Gypsy retrotransposons are grouped into CRM, Galadriel, Reina, Tcn1, Tekay (Del/Del1), Athila, Phygy and Tat (Metavirus). Gypsy lineages are further grouped into different branches according to the presence of a chromodomain, grouping together CRM, Galadriel, Reina, Tcn1, and Tekay (Del/Del1) lineages into the Chromovirus branch^27,28^. Moreover, previous studies have found that plant *Tcn1* sequences representatives share high similarity to that of *Cryptococcus neoformans*, which may be the result of a horizontal transfer from fungi, which have not been deeply studied^31,32^. So, to better understand the hierarchical classification and complicated pattern of evolution, compiling a multi-species, comprehensive large-scale LTR-RT dataset is of great necessity.

With the advent of modern sequencing technologies and the availability of genomic resources for many organisms, different TEs databases have become available. These databases can be divided into two main types of focus: 1) analysis and classification of TE based on their phylogenetics (per lineage and protein domain), such as GyDB^28^ and REXdb^27^ and 2) identification and characterization of TEs in specific species, such as GrTEdb^33^ and DPTEdb^34^. However, there is no database for systematic and unified processing of LTR-RTs of plants, including Rhodophyta, Chlorophyta, Bryophytes, Pteridophyta, Gymnosperm, and Angiosperm. To make better use of and compare LTR-RTs in plants, it is necessary to establish a dataset containing these plants phylum to annotate LTR-RTs comprehensively and uniformly.

**Fig. 1:**
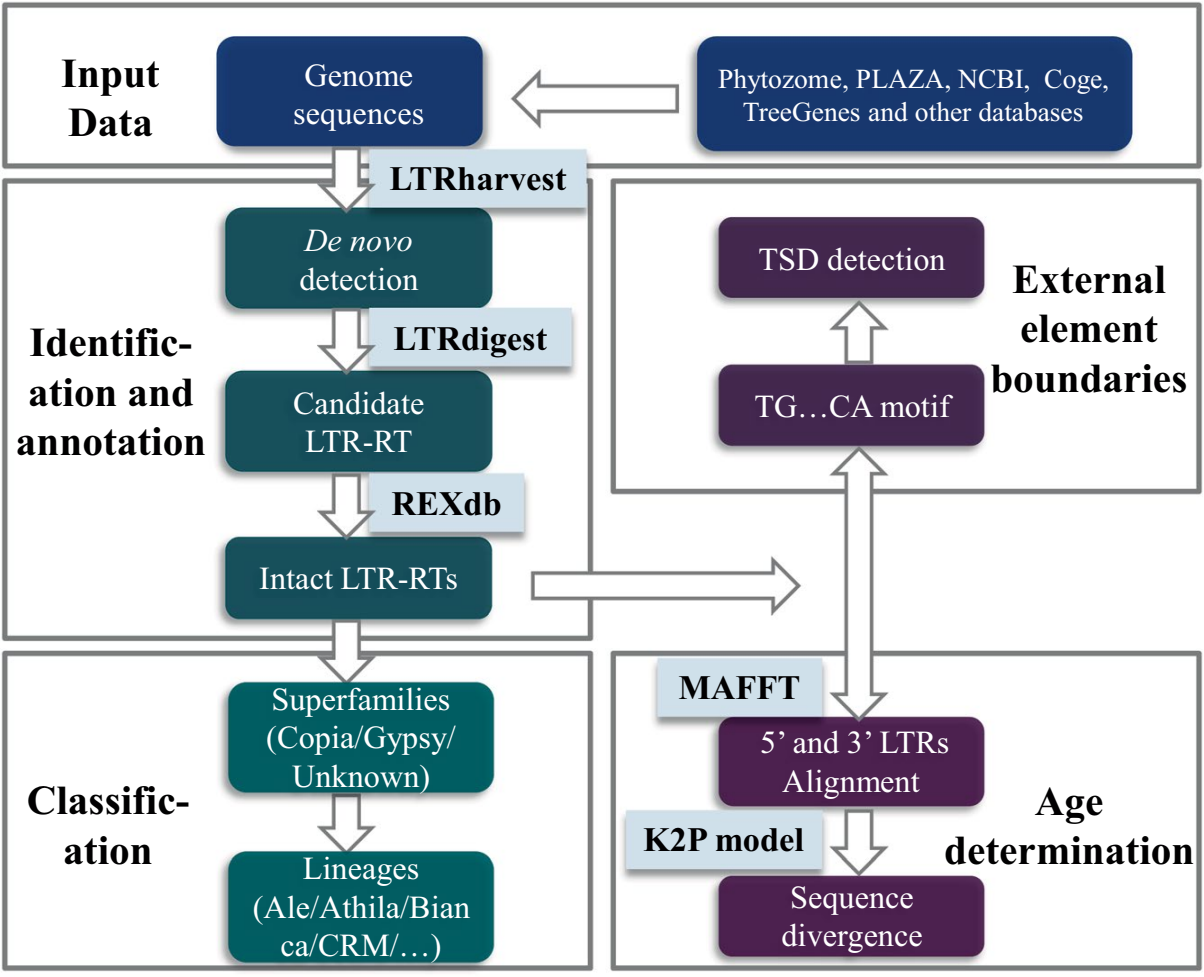
Schematic diagram illustrating the overall process of the intact LTR-RTs characterization in plant genomes. The top section shows the data sources of plant genomes, and the following four different modules represent different analyses.

The LTR-RTs dataset presented here has been established following the schematic shown in Fig. 1. In this framework, a comprehensive annotated dataset of a total of 2,593,685 intact LTR-RTs from 300 plant genomes is presented. This dataset contributes to broadening the availability of information useful for the classification of LTR-RTs by: 1) identifying all intact LTR-RTs from diverse whole plant genomes; 2) accomplishing the functional annotation (coding domains including GAG, AP, INT, RT and RH) and classification of intact LTR-RTs; and 3) determining the age distribution of intact LTR-RTs with Kimura two-parameter model. Further details of dataset generation and contents are also provided. The dataset released in this study covers a wide breadth of highly complex plants and is expected to provide a useful resource of LTR-RTs.

## Methods

### Genomic data collection

A total of 301 plants genome assemblies were collected from multiple comprehensive databases such as Phytozome (v12, https://phytozome.jgi.doe.gov/pz/portal.html)^35^, PLAZA (https://bioinformatics.psb.ugent.be/plaza/), NCBI GenBank (https://www.ncbi.nlm.nih.gov/genome/), CoGe (https://genomevolution.org), TreeGenes (https://treegenesdb.org/Drupal) and other individual genome databases. In this study, the collected genomic data represent 93 families of 46 orders. Our taxon sampling includes 2 Rhodophyta, 5 Chlorophyta, 3 Bryophytes, 4 Pteridophyta, 10 Gymnosperm, and 277 Angiosperm species. Detailed information (species, genus, family, order names, links to the published genome articles and URLs for the species genome assemblies) is provided in Supplementary Table 1.

### Identification of LTR-RTs

All 301 plant genomes were searched for the *de novo* detection of LTR-RTs using LTRharvest^36^ and LTRdigest^37^ programs. We required that an LTR-RT is separated by 1 to 15 kb from other candidates and flanked by a pair of putative LTRs ranging from 100 to 3,000 bp with similarity > 80%. We obtained 12,829,207 candidate LTR-RTs from the 301 plant genomes, except for *Genlisea aurea*, a carnivorous plant with an unusually small genome size of 63.6 Mb, one of the smallest known among all higher plants. The genome of *G. aurea* was investigated for LTR-RT content using the default settings in RepeatMasker v4.0.7^38^ with the RepBase version 20170127 library^39^, and we found a few fragmented LTR-RTs but potentially no full-length intact LTR-RTs, which is consistent with a previous study^40^. Further, all the internal sequences of candidate LTR-RTs were annotated by aligning the Gag-Pol protein sequences to the reference library REXdb (http://repeatexplorer.org/?page_id=918)^27^. Alignment was performed, using LAST v983 (http://last.cbrc.jp)^41^, with the following parameters: “-L 10 -m 70 -P BL80 -e 80”. Those LTR-RTs containing alignments with the domains of “GAG” (Capsid protein), “AP” (Aspartic proteinase), “INT” (Integrase), “RT” (Reverse transcriptase), and “RH” (RNase H) were considered as intact LTR-RTs. Finally, the resulting dataset consisted of 2,593,685 intact LTR-RTs from 300 plant genomes.

### Reconstruction of LTR-RTs superfamilies and lineages

Depending on the order and similarity of protein domains in the *Pol* gene, the identified intact LTR-RTs were mainly classified into Copia and Gypsy superfamilies. We found some unclassified elements (8,682) because there were multiple Gag-Pol protein sequences that occurred inside the LTR-RTs. The Copia and Gypsy sequences were further grouped into 18 lineages based on their phylogenetic relationships and structural features of the elements within the REXdb database^27^.

### TGCA and TSD detection

In plants, LTRs are typically flanked by 2-bp palindromic motifs, commonly 5′-TG…CA-3′, with some rare exceptions and TSD is a small exact repeat that may occur at the insertion site. They normally show a high sequence identity but may have acquired mutational variation over evolutionary processes. The two nearly identical LTR sequences of LTR-RTs were flanked by TSDs of usually 4–6 bp. We determined LTR ends (TG at the 5′ end of 5′ LTR and CA at the 3′ end of 3′ LTR) and then searched for how often the next 4, 5 and 6 bp can be used to identify their direct orientation precisely flanking each side of the LTR ends.

### Age determination of LTR-RTs with Kimura distance*-*based calculation

To assess the evolutionary role of LTR-RTs, it is important to estimate when LTR-RT integration into the genome took place. The insertion of an LTR-RT creates a pair of LTRs with identical sequences at the two breakpoints, and subsequent accumulation of mutations between the pair of LTRs of one LTR-RT can be used as a measure of the elapsed time after the insertion. Here, we used nucleotide sequence divergence of a pair of LTRs as a proxy for LTR-RT’s insertion age. MAFFT^42^ with default parameters was used to align the 5′ and 3′ LTRs of each intact LTR-RT. Sequence divergence was then calculated using Kimura two-parameter (K2P) model^43^. Insertion times can be converted into million years given a lineage-specific synonymous substitution rate per site per year.

## Data Records

**Fig. 2:**
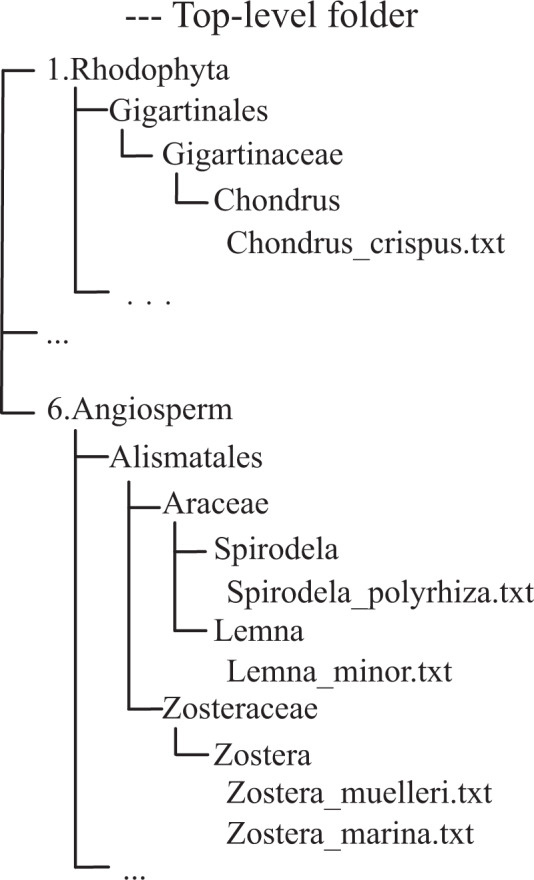
Illustration of the data structure.

The dataset containing the intact LTR-RTs information from 300 plant genomes resulting in 2,593,685 intact LTR-RTs with diverse structural, functional annotation, age determination, and classification information is available from the Figshare Repository^44^. The organization of the data collection is illustrated in Fig. 2. The top-level folder contains six sub-folders containing the intact LTR-RT data from Rhodophyta, Chlorophyta, Bryophytes, Pteridophyta, Gymnosperm, and Angiosperm and each sub-folder is further subdivided according to the plant order, family, and genus assignment.

### File format

All data are stored in plain text (txt) format. The file is named as “X.txt”, where “X” is a species’ scientific name. Each text file includes the structure information and divergence of intact LTR-RTs detected for a specific plant genome. Table 1 summarizes the keys for the metadata.

**Table 1:** Description of metadata keys for the plain text (.txt) files.

Key	Type	Description
**Species**	string	Species name
**LTR_ID**	string	ID of intact LTR-RTs
**Chromosome**	string	Chromosome of intact LTR-RTs
**Start**	int	Start position of domain in intact LTR-RTs
**End**	int	End position of domain in intact LTR-RTs
**Domain**	string	Type of domain in intact LTR-RTs
**Length(bp)**	int	Length of intact LTR-RTs
**Superfamilies**	string	Type of superfamilies
**Lineages**	string	Type of Lineages
**Divergence**	float	Sequence divergence of intact LTR-RTs

### Graphical representation of the dataset

**Fig. 3:**
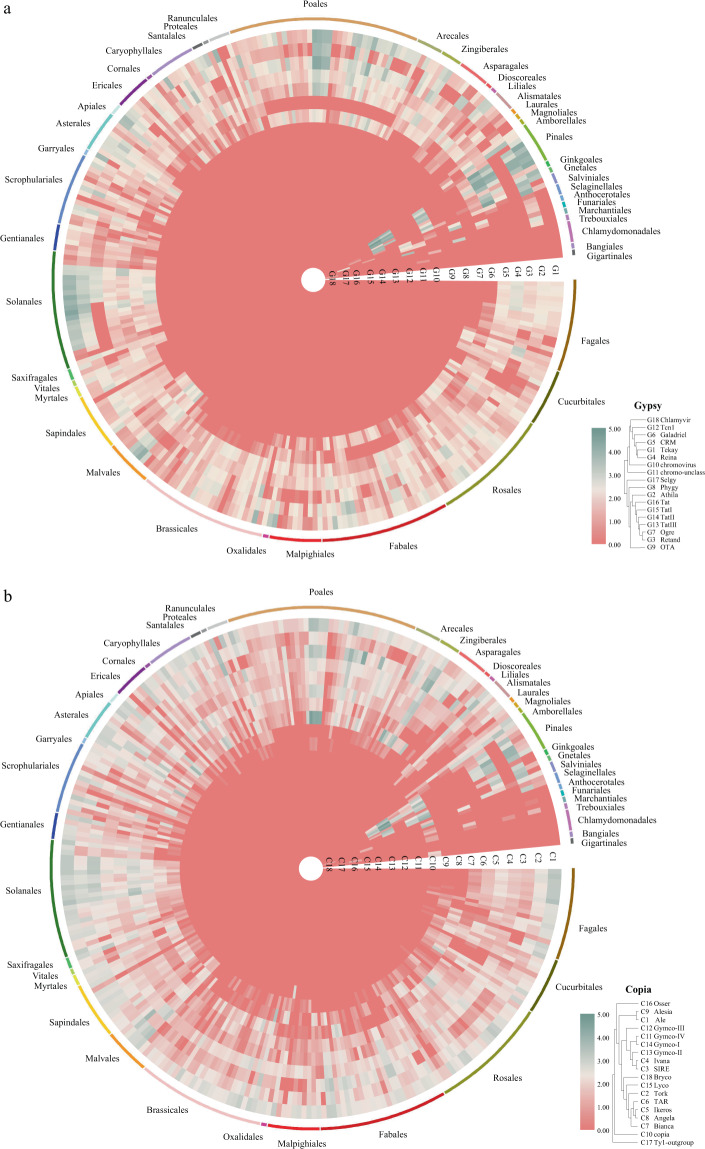
Intact LTR-RT (Gypsy and Copia) occupation of plant genomes. Resolved intact LTR-RT lineages were identified in 300 plant genomes of diverse systematic assignment. The presence of intact LTR-RT lineages is shown as heatmap determined by the log-transformed (log10) value of the intact LTR-RT copy number. The realized phylogenetic relationship of LTR-RT lineages^24^ is shown in the bottom right corner. (**a**) Gypsy superfamily. (**b**) Copia superfamily.

Figure 3 presents the distribution of intact LTR-RT lineages of the studied 300 plant genomes.

**Fig. 4:**
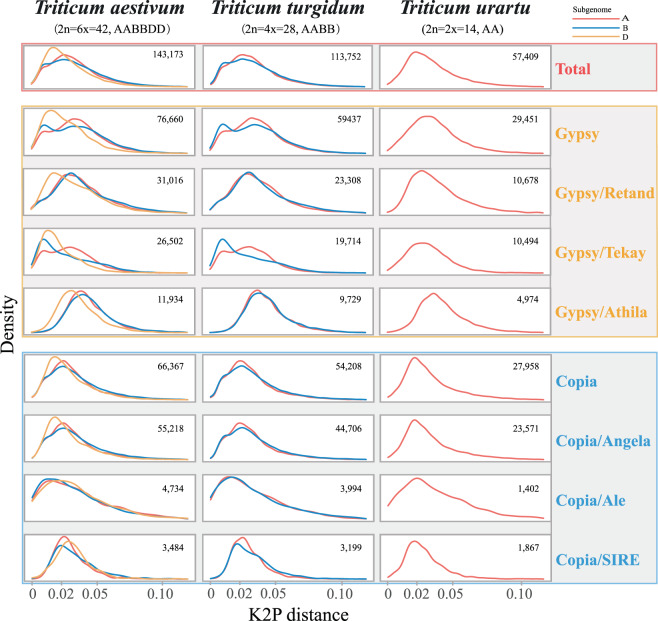
Density map of age distribution of intact LTR-RTs in representative *Triticum* species. For each species, intact LTR-RTs were grouped in both superfamilies and lineages (only the first few dominant lineages are shown here). The proportion of intact LTR-RTs of each specific age bin is shown, and subgenomes (A, B and D) from three *Triticum* species are colored red, blue and yellow, respectively.

We further analyzed sequence divergence measured by K2P distance and intact LTR-RT activity pattern of representative wheat (*Triticum*) species, one of the most important cereal grain crops (Fig. 4**)**. Differences in historical proliferation dynamics were shown among different LTR-RT subfamilies of different subgenomes in different plants with different ploidy.

## Technical Validation

To validate the dataset, we compared the intact LTR-RTs annotation acquired by sequence similarity and the *de novo* (used in this study) method. We chose rice (*Oryza sativa*. ssp. *japonica*) as a representative species for quality control as its genome is intensively examined and well annotated. A manually curated LTR-RTs library including 897 elements of rice was prepared in a previous study^45^. This library included known LTR-RT elements like *RIRE1* (named as *Angela* in our dataset), *RIRE2* (*Retand*), *RIRE3* (*Tekay*), *CRR* (*CRM*) and *Truncator* (*Tekay*). Next, we annotated 897 LTR-RT sequences against the REXdb database^27^ using LAST software^41^. Among them, 247 sequences possessed complete Gag-Pol protein sequences, which were considered as intact LTR-RTs. These candidate intact LTR-RTs sequences were then mapped to the *Oryza sativa*. ssp. *japonica* genome (Nip-BRI) using RepeatMasker software^38^ with default parameters. Finally, we acquired in total the 3,002 intact LTR-RTs, of which 2,332 elements were consistent with our results (2,941 elements) obtained by *de novo* method. This comparison confirmed the reliability of our dataset.

**Fig. 5:**
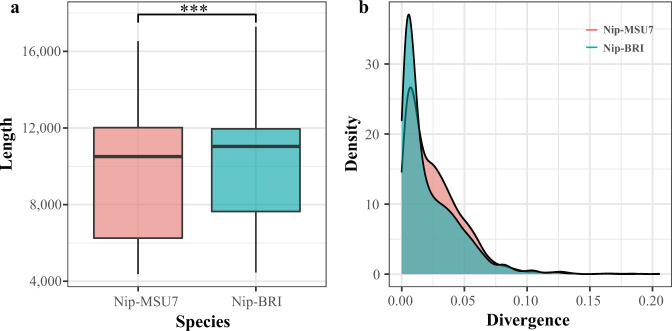
Comparison of LTR-RT length and insertion time identified in two rice genome assemblies. (**a**) Difference of LTR-RT length between Nip-BRI and Nip-MSU7. *** shows a *P*-value of less than 0.001. (**b**) Insertion time of LTR-RT estimated by sequence divergence of the two LTRs in Nip-BRI and Nip-MSU7.

The differences in LTR-RT content, length and age structure in *Oryza sativa*. ssp. *japonica* may be influenced by assembly quality. A total of 2,941 LTR-RTs was detected in *O. sativa*. ssp. *japonica* (Nip-BRI)^46^, an updated assembly from long-reads sequencing, compared with 2,636 in Nip-MSU7^47^, a short-read based assembly, and also, no LTR-RT with multiple Gag-Pol was identified in the updated assembly (Table 2). Wilcoxon test showed that the LTR-RT length identified in the Nip-BRI was significantly longer than that in the Nip-MSU7 (Fig. 5a). We further found that the insertion time of an LTR-RT estimated assembly by sequence divergence of the two LTRs in the Nip-BRI was significantly younger than that in the Nip-MSU7 (Wilcoxon test, p < 2.22e-16), indicating that many recently inserted LTR-RTs were unidentified in the Nip-MSU7 genome generated by short-reads sequencing (Fig. 5b). These findings suggest that high-quality assembled genomes obtained by long-read sequencing technology are critical to the identification and classification of LTR-RTs.

**Table 2:** Comparison of LTR-RT annotated in two *Oryza sativa* genome assemblies.

Key	Nip-BRI	Nip-MSU7
**Assembly size/Mb**	380.70	373.25
**Contig N50/Mb**	17	7.7
**Number of Gap**	18	905
**Number of Intact LTR-RTs**	2,941	2,636
**Length/Mb**	29,589,668	25,529,468
**Percent/%**	7.78	6.84
**Number of Intact LTR-RTs with multiple Gag-Pol**	0	2

Several databases describing TE reference sequences have been published. The Repbase Update contains consensus sequences of LTR-RT superfamilies and lineages^39^, but lacks information on internal structure. The Gypsy database (GyDB) compiles LTR-RTs and Retroviridae-like elements^28^, but the metadata of Gypsy/Copia lineages is not comprehensive. REXdb divides Copia and Gypsy retrotransposons into 16 and 14 lineages, respectively, based on the conserved polyprotein domains^27^, but is derived from a relatively small sampling of sequences from 80 species. In the current study, we compile a dataset of LTR-RTs in plants to further enable comparative and evolutionary studies in plants. The dataset is dedicated to the identification and classification of intact LTR-RTs in 300 plant genomes using comprehensive and unified annotation approaches. Furthermore, it provides information on age distribution of intact LTR-RTs with Kimura two-parameter model.

## Usage Notes

We envision many possible uses for this dataset, especially for the study of the origin, amplification, functional impact, and evolutionary dynamics of LTR-RTs among species and to encourage its use for evaluating the impact of LTR-RTs on host genomes, and to analyze the potential interaction between LTR-RTs and protein-coding genes such as:

1. *Solo LTRs and truncated LTR-RTs detection*. Unpaired LTRs (solo LTRs and truncated LTR-RTs) could further be determined based on the information of intact LTR-RTs^48^, since the ratios between intact LTR-RTs and solo LTRs have been used to estimate purge rates (removal rate). Removal rate could help to further research on why different plant genomes have distinct removal rate and understand molecular mechanisms of DNA amplification and removal.
2. *LRT-RT insertion and associated factors.* LTR-RT insertion times can be used to reveal the dynamics of LTR-RTs and their impact on genome evolution. For available sequence divergences of each species, insertion times could be converted into million years when given a lineage-specific synonymous substitution rate per site per year. When an LTR-RT’s proliferation time is determined, it could further be associated with historical processes, like environmental changes, mating transition, historical hybridization, and polyploidization/diploidization, so as to reveal the potential biological mechanism.
3. *LTR-RT’s expression and its functional impact.* Quantitative expression of LTR-RTs could be performed by RNA sequencing or RT-qPCR in plant tissues. In addition, RNA-seq data can be used to dissect the effect of LTR-RT insertions and analyze the expression from the targeted genomic region. Furthermore, R packages, like TEtranscripts, could be used to analyze TEs, including LTR-RTs in differential expression analysis of RNA-seq data^49^. The analysis of LTR-RTs expression could help understanding how these elements affect cell function to preserve specific tissues physiology and homeostasis in the plant.
4. *LTR-RT’s involvement in gene regulation.* DNA methylation and hydroxymethylation could be measured to understand the genome-wide epigenetic regulation of LTR-RTs. Additionally, several transcription factors were found to have their binding sites frequently located within various types of TEs, particularly LTR-RTs for ChIP-seq data, potentially leading to cell-specific gene regulation^50,51^. LTR-RT changes in adjacent gene regulation could further infer whether the contribution to plant fitness is positive, neutral, or negative.
5. *LTR-RTs derived gene duplication.* Genes can be duplicated through an RNA intermediate in a process mediated by retrotransposons as functional retrocopies or retrogenes, and they are mostly flanked by LTR-RTs in plants. Our dataset could help identify retrogenes and related duplicates, thus can help further investigate their contribution to species-specific phenotypic variation. For example, *Sun* is involved in the morphological variation of the tomato fruit^52^.
6. *Lateral transfer of LTR-RTs.* Horizontal transfers (HTs) usually represent the transmission of genetic material between reproductively isolated species and could allow TEs to escape their original host by transposing into a new organism, ensuring their survival. However, although HTs are common in plants, studies of horizontal TE transfers (HTTs) remain scarce because of limited taxa sampling^53^. Our dataset is valuable for further study of HTTs based on a larger taxon sampling covering most major plant orders.
7. *LTR-RTs and genome size variation.* In flowering plants, changes in copy number of retrotransposons appear to be the main factor responsible for genome size differences between species, in addition to polyploidy. It is found that the maize genome is 3–4 times as large as the sorghum genome, which is mainly caused by the extensive proliferation of retrotransposons (especially LTR-RTs) after the divergence of the two species^54^. Differences in the activity of retrotransposon regulation mechanisms (the proliferation of LTR-RTs) or their deletion generation (removal rate of LTR-RTs mentioned above) between species could explain current genome size variation. The present dataset brings a starting point for further systematic investigation of LTR-RT’s roles in genome size variation.

## Supplementary information

Supplementary Table 1

## Data Availability

To prepare this dataset, we used LTRharvest and LTRdigest from genometools version 1.5.10 software^55^ and REXdb database (http://repeatexplorer.org/)^27^. The sources for the 301 plant genomes can be downloaded through the link provided in Supplementary Table 1 and scripts for intact LTR-RTs annotation are available at GitHub link (https://github.com/sszhou9/intact-LTR-RTs).
